# Emerging Therapies for Childhood Polycystic Kidney Disease

**DOI:** 10.3389/fped.2017.00077

**Published:** 2017-04-19

**Authors:** William E. Sweeney, Ellis D. Avner

**Affiliations:** ^1^Department of Pediatrics, Medical College of Wisconsin, Children’s Research Institute, Children’s Hospital Health System of Wisconsin, Milwaukee, WI, USA

**Keywords:** childhood PKD, therapy, tolvaptan, combination therapy, multi-kinase inhibitors

## Abstract

Cystic kidney diseases comprise a varied collection of hereditary disorders, where renal cysts comprise a major element of their pleiotropic phenotype. In pediatric patients, the term polycystic kidney disease (PKD) commonly refers to two specific hereditary diseases, autosomal recessive polycystic kidney disease (ARPKD) and autosomal dominant polycystic kidney disease (ADPKD). Remarkable progress has been made in understanding the complex molecular and cellular mechanisms of renal cyst formation in ARPKD and ADPKD. One of the most important discoveries is that both the genes and proteins products of ARPKD and ADPKD interact in a complex network of genetic and functional interactions. These interactions and the shared phenotypic abnormalities of ARPKD and ADPKD, the “cystic phenotypes” suggest that many of the therapies developed and tested for ADPKD may be effective in ARPKD as well. Successful therapeutic interventions for childhood PKD will, therefore, be guided by knowledge of these molecular interactions, as well as a number of clinical parameters, such as the stage of the disease and the rate of disease progression.

## Autosomal Recessive Polycystic Kidney Disease (ARPKD)

Autosomal recessive polycystic kidney disease (OMIM #263200) is characterized by renal cysts and hepatobiliary dysgenesis and is a substantial cause of morbidity and mortality in children (1, 2). ARPKD is caused by mutations in the *PKHD1* gene which encodes a protein known as fibrocystin or polyductin (FPC), and both the gene and protein interact with the autosomal dominant polycystic kidney disease (ADPKD) genes and proteins.

## Autosomal Dominant Polycystic Kidney Disease

Autosomal dominant polycystic kidney disease is one of the most common genetic disease affecting 1/400 to 1/1,000 individuals worldwide. ADPKD is generally a late-onset, systemic disease characterized by bilateral, progressive enlargement of focal cysts occurring in all nephron segments with variable extrarenal manifestations (3).

Autosomal dominant polycystic kidney disease was originally thought to be caused by mutations in two genes; *PKD1* (on chromosome 16p13.3) (OMIM #173900) and *PKD2* (on chromosome 4q21) (OMIM #173910). These genes encode the protein polycystin 1 (PC1) and polycystin 2 (PC2), respectively.

There has always been a nagging suspicion that at least one additional disease causing gene was as yet undiscovered because there has always been a small number of genetically unresolved families (GUR) that did not link to either locus (4–6). Recent reexamination of these GUR families demonstrated that mutations *in GANAB* (OMIM 104160) encoding the glucosidase II subunit α on chromosome 11q12.3 cause a mild form of ADPKD and autosomal dominant polycystic liver disease of varying severity, most likely due to defects in PC1 maturation (7).

Childhood ADPKD may be indistinguishable from ARPKD, and histological or genetic analysis may be necessary to differentiate the two (2, 8). The prevalence of pediatric patients with ADPKD in our polycystic kidney disease (PKD) clinic is approximately equivalent to the number of ARPKD patients, and both are significant sources of morbidity and mortality in children. The interaction between the genes, proteins, and overlapping cystic phenotypes suggests that therapeutic interventions and lessons learned from clinical trials in ADPKD can be applied to patients with ARPKD.

## Pathophysiology and Translational Implications

### Cellular Pathophysiology

Cyst formation and progressive growth is a complex dynamic process with multiple interacting signaling components that all contribute to disease, but never act autonomously. The early investigations of PKD focused on fundamental phenotypic changes that would be necessary for a normal renal tubular epithelial cell to become a cystic epithelial cell. Normal renal epithelial cells changed from a mature, differentiated, non-proliferative, absorptive cell to a partially de-differentiated secretory cell characterized by specific polarity defects and increased rates of proliferation (9).

**Figure 1:**
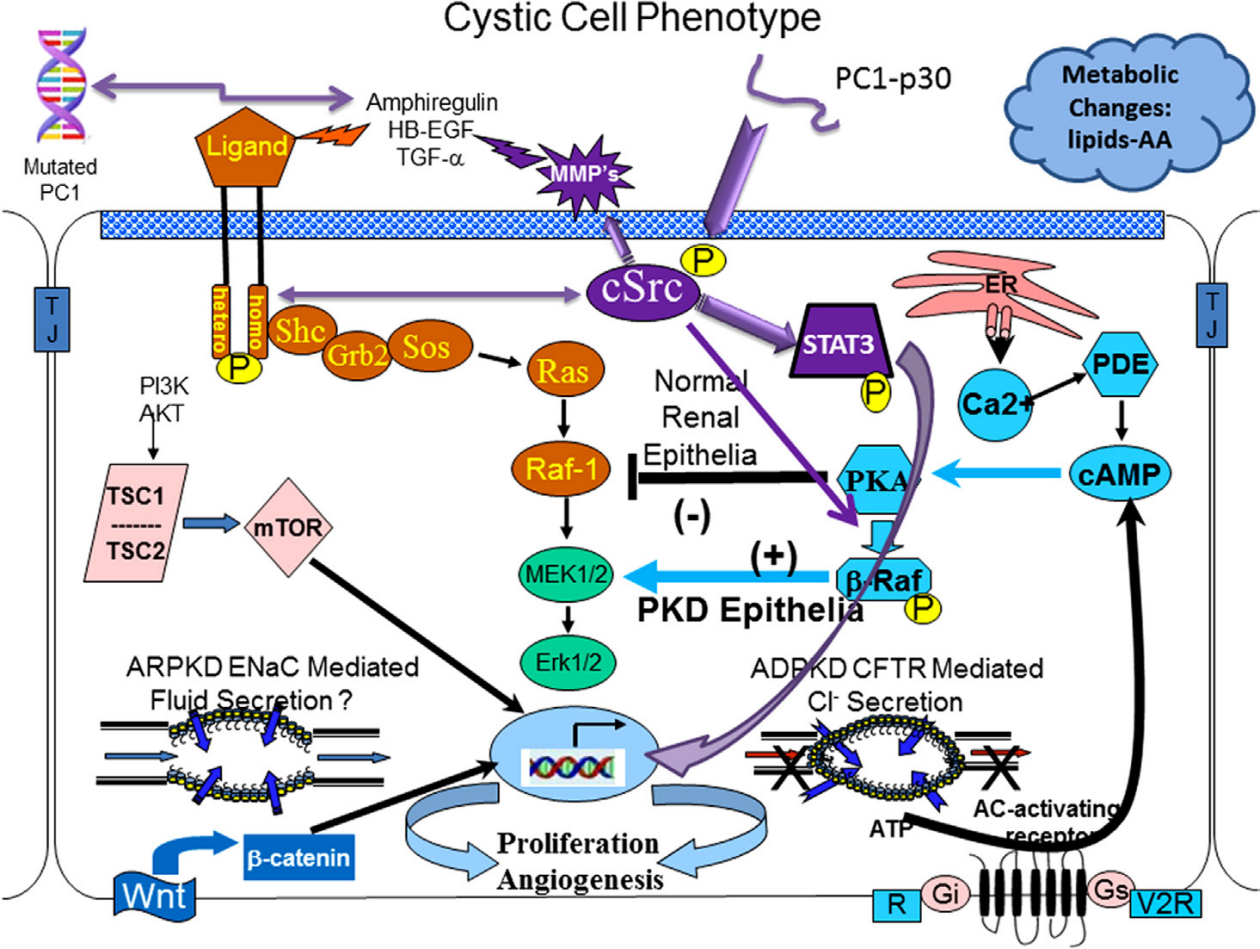
**The cystic cellular phenotype**. This cartoon is an abridged composite of the abnormal signal transduction pathways reported to be active in polycystic kidney disease (PKD). Two main conduits that lead to unchecked proliferation are (1) the EGFR axis (orange path) and (2) a G-protein axis (aqua blue path) that leads to increased cyclic adenosine monophosphate (cAMP) and a switch in phenotypic response of renal epithelia to cAMP. The pathways suggest the following: in autosomal recessive polycystic kidney disease (ARPKD), an apical EGFR results in the axis becoming active resulting in reciprocal phosphorylation of the non-receptor tyrosine kinase cSrc (purple); in autosomal dominant polycystic kidney disease (ADPKD) a mutated polycystin 1 (PC1) leads to increase amphiregulin, activating EGFR, resulting in increased cSrc phosphorylation; in both ARPKD and ADPKD, cSrc activity (purple) alters the cellular response of cAMP resulting in proliferation; in addition, in ADPKD the cytoplasmic tail, PC1-p30 is overexpressed leading to acSrc-dependent activation of STAT3 by tyrosine phosphorylation. EGFR and cAMP signaling amplify the activation of cSrc/STAT3 by PC1-p30. Targeting proliferation will always be a requirement to effectively slow the progression of PKD and prevent the need for renal replacement therapy. Targeting single molecules that bridge both pathways (such as cSrc) is a logical approach to get maximum effectiveness with minimal dosing, thereby limiting toxicity. Pharmacological inhibition with a single compound that targets multiple pathways (such as a multi-kinase inhibitor—tesevatinib) should provide a similar benefit. However, no single compound will provide lifetime effectiveness. Effective therapy will require multiple compounds administered in a disease stage-specific manner that will need to be individualized, accounting for variations in disease severity and rate of progression.

These led to the identification of a myriad of signaling molecules and signaling pathways that were found to be abnormal in cystic epithelium. Collectively, these changes define what is referred to as the “cystic phenotype.” Figure 1 is a cartoon that includes some but not all of the aberrant signaling pathways that constitute this cystic phenotype. The precise mechanisms by which mutated PKD proteins disrupt normal signaling and cause renal cyst are not entirely understood but significant progress in understanding the cellular events have been made.

*In vitro* and *in vivo* experimental models have been used to identify fundamental pathogenic features in human PKD and are thought to be pathogenic in both ARPKD and ADPKD. These include the following:

- aberrant intracellular levels of the second messenger, cyclic adenosine monophosphate (cAMP), coupled with decreased intracellular calcium increasing both proliferation and secretion;
- abnormalities in expression and localization of the ErbB or epidermal growth factor (EGFR)—family of receptors and ligands (EGFR axis), leading to increased proliferation;
- abnormal activity of cSrc (p60Src), a non-receptor tyrosine kinase that serves as a critical mediator of cross talk between the EGFR axis and G-protein-cAMP pathways. In addition, cSrc interactions with the cleaved C-terminal PC1 tail (PC1-p30) activate the transcription factor STAT3;
- activation of mammalian target of rapamycin (mTOR) signaling;
- alterations in cell–cell and cell–matrix interactions;
- changes in interstitial macrophages that leads to progressive interstitial fibrosis.

Clinical trials have to date been largely based on targeting these complex cellular signaling features of a cystic cell. Therefore, a brief discussion of each and the evidence supporting the pathogenic features listed above are included below. A brief discussion of the results of clinical trials to date will follow. A list of current and completed clinical trials can be found at http://clinicaltrials.gov/.

### cAMP-Mediated Proliferation and Secretion

The cAMP-dependent pathway is a G-protein-coupled receptor signaling pathway. Increased intracellular cAMP levels in a normal renal epithelial cell elicit a response that reduces MAPK activity resulting in a decreased rate of cellular proliferation. However, under conditions of low levels of intracellular calcium, a cSrc-dependent phosphorylation of β-Raf, allows the cell to bypass Raf-1and increase ERK phosphorylation and subsequent cell proliferation (see Figure 1) (10, 11). This also changes the normally absorptive renal epithelia cell to a secretory renal epithelial cell which contributes significantly to the progressive enlargement of cysts in ADPKD (12).

Therapeutically, increased cAMP is reduced by vasopressin R2 receptor antagonists, such as tolvaptan or somatostatin (and its long-acting analogs such as lanreotide), that inhibit adenylate cyclase resulting in reduced cAMP levels (13, 14).

Tolvaptan (Jinarc^®^) has been tested in human patients with ADPKD and the results have been encouraging. Tolvaptan slowed the increased in total kidney volume and slowed the decline in loss of renal function (15, 16). Tolvaptan has been approved in Europe, Canada, and Japan for treatment of ADPKD. However, it has not been approved in the USA due to persisting concerns regarding therapeutic effectiveness and concerns regarding potential hepatic injury.^1^ Tolvaptan is, therefore, not currently recommended for the treatment of childhood PKD.

### EGFR (ErbB) Axis

An abundance of evidence demonstrates that the epidermal growth factor receptor (EGFR) and other ErbB receptors and their ligands (the EGFR axis) are important mediators of renal cystic epithelial proliferation in PKD. In human ADPKD and ARPKD and rodent models of PKD, renal cystic epithelia display overexpression and mislocalization of one or more ErbB receptors to the apical cell surface instead of the customary basolateral localization seen in the normal human kidneys (17–19). These apically expressed ErbB receptors are functional and capable of generating a proliferative signal *in vitro* (20). Preclinical studies have demonstrated that *in vitro* and *in vivo* inhibition of ErbB receptor tyrosine kinase activity with tyrosine kinase inhibitors (21, 22) or genetic manipulation (23) and/or reduced ErbB ligand availability (24) significantly reduced cyst formation and enlargement.

Recent studies have provided a direct link between PC1 and the EGFR ligand amphiregulin. Studies revealed that the promoter activity increased and established that cells with a mutated PC1, a reduced level of PC1, and primary cystic cells isolated from ADPKD kidneys exhibit increased amphiregulin expression (25, 26). In addition, microarray profiling of human ADPKD cells and a conditional mouse model (*Cre*;*Pkd1^del2-11,lox^*) found that ErbB4 activation was a major driver of cellular proliferation in ADPKD and may well be a biomarker of disease progression (27).

### cSrc

cSrc is a critical intermediate that connects both the cyclic AMP and EGFR pathways and, therefore, plays a critical role in integrating signaling in normal and cystic epithelium. In ADPKD, *PKD1* mutations give rise to increased production of amphiregulin that in turns activates (phosphorylates) the EGF receptor resulting in a reciprocal phosphorylation (activation) of cSrc. Phosphorylated cSrc activates β-Raf, which alters the response of renal epithelia to cAMP from a normally antimitotic to a pro-proliferative phenotype. Additionally, activated cSrc interacts with PC1-p30, a proteolytic fragment of the PC1 cytoplasmic tail, resulting in STAT3 phosphorylation and further increased proliferation (28, 29). This cSrc-mediated activation of STAT 3 is augmented by increased activity of the EGFR axis and increased cAMP, thereby promoting even greater proliferation of tubular epithelium and cyst enlargement (29, 30).

These interactions forecast a pathologic proliferation pathway where mutated PC1 increases amphiregulin expression, resulting in activation of ErbB receptors and reciprocal phosphorylation of cSrc. Phosphorylated cSrc integrates and amplifies proliferative signals from EGFR and cAMP and together with the PC1 cytoplasmic tail, PC1-p30, activates STAT3 which leads to even further intensification of the proliferative signals (29).

Bosutinib, a cSrc inhibitor, has undergone clinical trials in ADPKD. Although very effective in reducing total kidney volume, it did not effect changes in renal function.^2^

### Mammalian Target of Rapamycin

The mTOR pathway integrates signals from growth factors (including EGFR), G-protein coupled receptors (which generate cAMP), cellular energy levels, nutrient status, and stress conditions to stimulate protein synthesis and cell growth through activation (phosphorylation) of S6K1 and eIF4E (31, 32). In human ADPKD and ARPKD and a variety of animal models, cyst-lining epithelium demonstrates increased activity of mTOR (32–35). The mTOR inhibitors rapamycin and everolimus have been tested in human clinical trials and were found to be ineffective in slowing total kidney volume or preventing loss of renal function (36, 37).

It is impossible to imagine how a single compound could provide lifetime effective therapy even if started early in the disease process. However, the identification of pathological cellular events provides a starting point for building future therapeutic interventional strategies for childhood PKD. Challenges including the focal nature of cyst formation and the large variation in the phenotypic expression of ARPKD and ADPKD are not trivial. The substantial intra-familial phenotypic variability seen in both ARPKD and ADPKD suggests that complex factors which influence or direct the timing and severity of disease are operative.

### Molecular Pathophysiology

Improved molecular techniques and increased specificity in producing targeted gene mutations has allowed development of orthologous rodent models with conditional and hypomorphic mutations in *Pkd1, Pkd2*, and *Pkhd1*. Studies in such models which more accurately reflect the human disease have yielded unexpected results regarding mechanisms of cyst development and enlargement in PKD. Advances in sequencing, in concert with improved methods to produce animal models with specific mutations including those found in PKD patients, allow suspected mechanistic processes to be directly tested. These new insights along with lessons learned from the original clinical trials will lead to novel therapeutic interventions for PKD.

For example, in an intricate study, the genetic combination of five cystic disease models, including orthologous *Pkd1, Pkd2, Pkhd1*, and the two polycystic liver disease genes, *Prkcsh* and *Sec63*, resulted in different combinations of mutant alleles which allowed the functional relationships between these genes to be defined (38). These combinations demonstrated that (1) *Prkcsh* and *Sec63* mutations result in impaired biogenesis of PC1; (2) PC1 dosage modifies the severity of both ADPKD and ARPKD; (3) the threshold level of PC1 necessary for normal tubular morphology varies by nephron segment with collecting ducts being most sensitive; and (4) overexpression of *Pkd1* is capable of rescuing a mutant *Pkhd1* animal (38).

Most if not all of the numerous cystic kidney disease proteins, including PC1, PC2, and FPC, are found in primary cilia or basal body of cilia which led to the cilia theory and the term “ciliopathies” to cover any disease caused by a mutation in a protein that is localized to the cilia (25, 39). PC1 and PC2 are predicted to form a complex on the primary cilium creating a mechanosensor that transmit external signals such as flow, to the renal epithelial cell (40, 41).

In a similar experiment as that described above, mouse models with tissue-specific and inducible knockouts of *Pkd1* and *Pkd2* alone or in combination with knockouts of cilia proteins *Kif3a* and *Ift20* revealed that: disruption of cilia reduced cyst growth caused by loss of PC1 or PC2; simultaneous loss of PCs and either Kif3a or Ift20 resulted in milder disease severity than that seen when either PC protein was inactivated; and the length of time that intact cilia existed after the loss of PC’s increased disease severity (42). This suggests the existence of a cilia-dependent proliferative or cyst-promoting pathway that is inhibited by a normal PC1/PC2 complex (43).

The mechanosensing function of primary cilia was originally thought to result in increased calcium influx into the cell. In the absence of a normal PC1/PC2 channel, calcium levels in the cell fall and in the context of high cAMP levels, the cell phenotype becomes cystic. In a recent study, the primary cilia of multiple cell types including renal epithelial were shown not to transmit a calcium signal upon bending (44). The authors concluded that if cilia act as mechanosensor or a flow sensor, it does not occur through calcium signals.

The two-hit theory, proposed to explain the focal nature of cyst formation in PKD, stated that a somatic mutation or “second hit” in addition to the germ-line mutation was necessary for a cell to become cystic. Although this may explain some of the focal nature of cyst formation in ADPKD, other factors have been shown to influence disease progression and severity. On a cellular level, these include: the developmental timing of *PKD1* inactivation (45, 46); reduction in functional PC1 dosage (38, 47, 48); differences in sensitivity to PC1 dosage (48); and the proximity effect, where a cystic cell or nephron creates a “snowball effect” triggering cyst development in neighboring cells or nephrons (49).

On a molecular level, a number of factors demonstrate that complex inheritance patterns influence disease severity in both ADPKD and ARPKD. These include hypomorphic or incompletely penetrant alleles (50); *PKD1* or *PKD2* homozygosity (47); compound heterozygosity (51); trans-heterozygosity (52); somatic and germ-line mosaicism (53); epigenetic regulators (54–57); genetic modifiers (58); co-inheritance of a *PKD1* or *PKD2* mutation and an additional cyst-causing gene such as *HNF1*β (59, 60) or the tuberous sclerosis 2 gene (61); and alternative splicing of *Pkhd1* that produces transcripts with distinct expression patterns and function (62).

### Clinical Trials and Lesson Learned

The diagnosis of childhood PKD is no longer the terminal diagnosis as was once considered. For children with ARPKD, advances in neonatal critical care and renal replacement therapy have allowed many to survive much longer than what was possible just a few decades ago. Insights into the development and treatment of congenital hepatic fibrosis and portal hypertension, a complication of the periportal fibrosis in ARPKD, have led to a better quality of life. Renal transplantation and dual organ transplantation provide an opportunity for these children to live a near normal life. Preimplantation genetic diagnosis holds the possibility of eliminating ARPKD from families who can afford the procedure and who are not ethically opposed (63). Preimplantation genetic diagnosis is also being evaluated for ADPKD spouses in China (NCT02948179).

As clinical trials to date have shown, targeting a single molecule of an aberrant pathway has not resulted in the reduction of disease burden as originally hoped for. However, even as the targeted therapeutic strategies develop more specificity they will always need to target (a) cell proliferation; (b) cAMP levels; and (c) fluid secretion. Proliferation is an absolute necessity for cyst formation and as such, it must be targeted and controlled for any therapy to be effective; how much and for how long remains unknown.

In the near future, the most promising therapies will target key signaling intermediates that integrate multiple pathways, such as cSrc (see Figure 1) (29, 64) and/or use a combination therapy approach where multiple compounds are used to target multiple pathways simultaneously or a single compound that targets multiple pathways such as a multi-kinase inhibitors such as tesevatinib (TSV) (65, 66). TSV, formerly known as KD-019, is currently undergoing testing in a Phase 2 clinical trial for ADPKD (NCT02616055) and has recently received FDA approval for a Phase 1 trial in young patients with ARPKD.

Successful therapies will require knowledge of the extent of the disease (i.e., how far along is the disease) when therapy begins and the rate of progression, will require multiple agents or a single agent that hits multiple targets, and the choice of targets will be stage specific and change as disease progresses.

There are a number of additional important factors to consider in developing therapies for childhood PKD. Therapies will increasingly focus on treating PKD in its earliest stages where they are likely to offer the maximum long-term benefit. Therapies that target abnormal proliferative pathways will need to be carefully monitored so that pathway activity is reduced to normal basal levels rather than completely shut off. Promising compounds may be modified to direct the molecule to the site of disease, making these therapies highly specific with low levels of toxicity (67). This specificity will accelerate the development of protocols for the ethical treatment of children with PKD where maximal quality-of-life benefit will come from early intervention. Epigenetic and dietary factors that slow or accelerate the progression of PKD will be discovered and adherence or avoidance of such factors may slow the rate of progression and eliminate the need for pharmacological intervention or renal replacement therapy for some.

The importance of genetic dosage, modifier genes, and somatic mutations in the clinical course of PKD in an individual patient provide compelling rationale for a personalized medicine approach. Personalized medicine will mean tailored approaches that modulate functional gene dosage and consider not only individual genotypes but also account for the response of the kidney to the disease and the unanticipated response of the kidney to therapy. This unanticipated response to therapy may be active in tolvaptan therapy, where patients treated with Jinarc^®^ were reported to have increased urinary shedding of heparin-binding EGF-like growth factor (HB-EGF) (68). In ARPKD and ADPKD cysts, cystic epithelia express EGFR or ErbB receptors on the apical side (urinary side) of the cell. The presence of HB-EGF, a powerful mitogen, in the urine may stimulate these apical receptors prompting a proliferative signal that would counter at least some of the reduced proliferation gained by decreasing cAMP levels with tolvaptan therapy. This suggests that a tyrosine kinase inhibitor against EGFR or an enzyme inhibitor to prevent HB-EGF processing or both, added to tolvaptan therapy may lead to greater benefit especially in terms of reduced total kidney volume.

The progress made in understanding the pathophysiology of PKD has been remarkable and despite the work still remaining, patients and parents can take heart that two clinical trials for children with PKD are underway: one for children with ADPKD [Tolvaptan (NCT02964273) Belgium and Italy] and one recently approved for the use of multi-kinase inhibitor, TSV in children with ARPKD.

## Author Contributions

WS and EA contributed equally to the conception and design of the manuscript; drafted the article and made critical revisions related to the intellectual content of the manuscript; and approved the final version of the article to be published.

## Conflict of Interest Statement

The authors declare that they have no conflicts of interest with respect to research, authorship and/or publication of this manuscript.

## Footnotes

^1^www.fda.gov/safety.

^2^http://ClinicalTrials.gov identifier NCT01233869.

## References

[1] Gunay-Aygun M Font-Montgomery E Lukose L Tuchman Gerstein M Piwnica-Worms K Choyke P Characteristics of congenital hepatic fibrosis in a large cohort of patients with autosomal recessive polycystic kidney disease. Gastroenterology (2013) 144:112–21. 10.1053/j.gastro.2012.09.056 23041322 PMC4162098

[2] Sweeney WE Jr Gunay-Aygun M Patil A Avner ED Childhood polycystic kidney disease. In: Avner ED Harmon WE Niaudet P Yoshikawa N Emma F Goldstein S, editors. Pediatric Nephrology. Heidelberg: Springer-Verlag (2016). p. 1103–54.

[3] Harris PC Torres VE. Polycystic kidney disease. Annu Rev Med (2009) 60:321–37. 10.1146/annurev.med.60.101707.125712 18947299 PMC2834200

[4] Daoust MC Reynolds DM Bichet DG Somlo S. Evidence for a third genetic locus for autosomal dominant polycystic kidney disease. Genomics (1995) 25:733–6. 10.1016/0888-7543(95)80020-M 7759112

[5] De Almeida S De Almeida E Peters D Pinto JR Tavora I Lavinha J Autosomal dominant polycystic kidney disease: evidence for the existence of a third locus in a Portuguese family. Hum Genet (1995) 96:83–8. 10.1007/BF00214191 7607660

[6] Turco AE Clementi M Rossetti S Tenconi R Pignatti PF An Italian family with autosomal dominant polycystic kidney disease unlinked to either the PKD1 or PKD2 gene. Am J Kidney Dis (1996) 28:759–61. 10.1016/S0272-6386(96)90261-9 9158217

[7] Porath B Gainullin VG Cornec-Le Gall E Dillinger EK Heyer CM Hopp K Mutations in GANAB, encoding the glucosidase IIalpha subunit, cause autosomal-dominant polycystic kidney and liver disease. Am J Hum Genet (2016) 98:1193–207. 10.1016/j.ajhg.2016.05.004 27259053 PMC4908191

[8] Sweeney WE Jr Avner ED. Diagnosis and management of childhood polycystic kidney disease. Pediatr Nephrol (2011) 26:675–92. 10.1007/s00467-010-1656-1 21046169

[9] Grantham JJ Cook LT Wetzel LH Cadnapaphornchai MA Bae KT. Evidence of extraordinary growth in the progressive enlargement of renal cysts. Clin J Am Soc Nephrol (2010) 5:889–96. 10.2215/CJN.00550110 20360307 PMC2863973

[10] Hanaoka K Guggino WB cAMP regulates cell proliferation and cyst formation in autosomal polycystic kidney disease cells. J Am Soc Nephrol (2000) 11:1179–87. 10864573 10.1681/ASN.V1171179

[11] Yamaguchi T Wallace DP Magenheimer BS Hempson SJ Grantham JJ Calvet JP. Calcium restriction allows cAMP activation of the B-Raf/ERK pathway, switching cells to a cAMP-dependent growth-stimulated phenotype. J Biol Chem (2004) 279:40419–30. 10.1074/jbc.M405079200 15263001

[12] Belibi FA Reif G Wallace DP Yamaguchi T Olsen L Li H Cyclic AMP promotes growth and secretion in human polycystic kidney epithelial cells. Kidney Int (2004) 66:964–73. 10.1111/j.1523-1755.2004.00843.x 15327388

[13] Gattone VH II Wang X Harris PC Torres VE. Inhibition of renal cystic disease development and progression by a vasopressin V2 receptor antagonist. Nat Med (2003) 9:1323–6. 10.1038/nm935 14502283

[14] Wang X Gattone V II Harris PC Torres VE. Effectiveness of vasopressin V2 receptor antagonists OPC-31260 and OPC-41061 on polycystic kidney disease development in the PCK rat. J Am Soc Nephrol (2005) 16:846–51. 10.1681/ASN.2004121090 15728778

[15] Torres VE Chapman AB Devuyst O Gansevoort RT Grantham JJ Higashihara E Tolvaptan in patients with autosomal dominant polycystic kidney disease. N Engl J Med (2012) 367:2407–18. 10.1056/NEJMoa1205511 23121377 PMC3760207

[16] Torres VE Higashihara E Devuyst O Chapman AB Gansevoort RT Grantham JJ Effect of tolvaptan in autosomal dominant polycystic kidney disease by CKD stage: results from the TEMPO 3:4 trial. Clin J Am Soc Nephrol (2016) 11:803–11. 10.2215/CJN.06300615 26912543 PMC4858477

[17] Du J Wilson PD. Abnormal polarization of EGF receptors and autocrine stimulation of cyst epithelial growth in human ADPKD. Am J Physiol (1995) 269:C487–95. 7653531 10.1152/ajpcell.1995.269.2.C487

[18] Orellana SA Sweeney WE Neff CD Avner ED. Epidermal growth factor receptor expression is abnormal in murine polycystic kidney. Kidney Int (1995) 47:490–9. 10.1038/ki.1995.62 7723235

[19] Pugh JL Sweeney WE Jr Avner ED. Tyrosine kinase activity of the EGF receptor in murine metanephric organ culture. Kidney Int (1995) 47:774–81. 10.1038/ki.1995.118 7752576

[20] Sweeney WE Avner ED. Functional activity of epidermal growth factor receptors in autosomal recessive polycystic kidney disease. Am J Physiol (1998) 275:F387–94. 9729511 10.1152/ajprenal.1998.275.3.F387

[21] Sweeney WE Chen Y Nakanishi K Frost P Avner ED. Treatment of polycystic kidney disease with a novel tyrosine kinase inhibitor. Kidney Int (2000) 57:33–40. 10.1046/j.1523-1755.2000.00829.x 10620185

[22] Sweeney WE Jr Hamahira K Sweeney J Garcia-Gatrell M Frost P Avner ED. Combination treatment of PKD utilizing dual inhibition of EGF-receptor activity and ligand bioavailability. Kidney Int (2003) 64:1310–9. 10.1046/j.1523-1755.2003.00232.x 12969149

[23] Richards WG Sweeney WE Yoder BK Wilkinson JE Woychik RP Avner ED. Epidermal growth factor receptor activity mediates renal cyst formation in polycystic kidney disease. J Clin Invest (1998) 101:935–9. 10.1172/JCI2071 9486961 PMC508642

[24] Dell KM Nemo R Sweeney WE Jr Levin JI Frost P Avner ED. A novel inhibitor of tumor necrosis factor-alpha converting enzyme ameliorates polycystic kidney disease. Kidney Int (2001) 60:1240–8. 10.1046/j.1523-1755.2001.00963.x 11576338

[25] Torres VE Harris PC Pirson Y. Autosomal dominant polycystic kidney disease. Lancet (2007) 369:1287–301. 10.1016/S0140-6736(07)60601-1 17434405

[26] Aguiari G Bizzarri F Bonon A Mangolini A Magri E Pedriali M Polycystin-1 regulates amphiregulin expression through CREB and AP1 signalling: implications in ADPKD cell proliferation. J Mol Med (2012) 90:1267–82. 10.1007/s00109-012-0902-3 22570239 PMC4028691

[27] Streets AJ Magayr TA Huang L Vergoz L Rossetti S Simms RJ Parallel microarray profiling identifies ErbB4 as a determinant of cyst growth in ADPKD and a prognostic biomarker for disease progression. Am J Physiol Renal Physiol (2017) 312:F577–88. 10.1152/ajprenal.00607.2016 28077374 PMC5504395

[28] Silva CM. Role of STATs as downstream signal transducers in Src family kinase-mediated tumorigenesis. Oncogene (2004) 23:8017–23. 10.1038/sj.onc.1208159 15489919

[29] Talbot JJ Song X Wang X Rinschen MM Doerr N Lariviere WB The cleaved cytoplasmic tail of polycystin-1 regulates Src-dependent STAT3 activation. J Am Soc Nephrol (2014) 25:1737–48. 10.1681/ASN.2013091026 24578126 PMC4116067

[30] Talbot JJ Shillingford JM Vasanth S Doerr N Mukherjee S Kinter MT Polycystin-1 regulates STAT activity by a dual mechanism. Proc Natl Acad Sci U S A (2011) 108:7985–90. 10.1073/pnas.1103816108 21518865 PMC3093515

[31] Wullschleger S Loewith R Hall MN TOR signaling in growth and metabolism. Cell (2006) 124:471–84. 10.1016/j.cell.2006.01.016 16469695

[32] Edelstein CL. Mammalian target of rapamycin and caspase inhibitors in polycystic kidney disease. Clin J Am Soc Nephrol (2008) 3:1219–26. 10.2215/CJN.05611207 18587045

[33] Shillingford JM Murcia NS Larson CH Low SH Hedgepeth R Brown N The mTOR pathway is regulated by polycystin-1, and its inhibition reverses renal cystogenesis in polycystic kidney disease. Proc Natl Acad Sci U S A (2006) 103:5466–71. 10.1073/pnas.0509694103 16567633 PMC1459378

[34] Zafar I Belibi FA He Z Edelstein CL. Long-term rapamycin therapy in the Han:SPRD rat model of polycystic kidney disease (PKD). Nephrol Dial Transplant (2009) 24:2349–53. 10.1093/ndt/gfp129 19321761 PMC2727300

[35] Shillingford JM Piontek KB Germino GG Weimbs T. Rapamycin ameliorates PKD resulting from conditional inactivation of Pkd1. J Am Soc Nephrol (2010) 21:489–97. 10.1681/ASN.2009040421 20075061 PMC2831854

[36] Serra AL Poster D Kistler AD Krauer F Raina S Young J Sirolimus and kidney growth in autosomal dominant polycystic kidney disease. N Engl J Med (2010) 363:820–9. 10.1056/NEJMoa0907419 20581391

[37] Walz G Budde K Mannaa M Nurnberger J Wanner C Sommerer C Everolimus in patients with autosomal dominant polycystic kidney disease. N Engl J Med (2010) 363:830–40. 10.1056/NEJMoa1003491 20581392

[38] Fedeles SV Tian X Gallagher AR Mitobe M Nishio S Lee SH A genetic interaction network of five genes for human polycystic kidney and liver diseases defines polycystin-1 as the central determinant of cyst formation. Nat Genet (2011) 43:639–47. 10.1038/ng.860 21685914 PMC3547075

[39] Pazour GJ. Intraflagellar transport and cilia-dependent renal disease: the ciliary hypothesis of polycystic kidney disease. J Am Soc Nephrol (2004) 15:2528–36. 10.1097/01.ASN.0000141055.57643.E0 15466257

[40] Nauli SM Alenghat FJ Luo Y Williams E Vassilev P Li X Polycystins 1 and 2 mediate mechanosensation in the primary cilium of kidney cells. Nat Genet (2003) 33:129–37. 10.1038/ng1076 12514735

[41] Nauli SM Rossetti S Kolb RJ Alenghat FJ Consugar MB Harris PC Loss of polycystin-1 in human cyst-lining epithelia leads to ciliary dysfunction. J Am Soc Nephrol (2006) 17:1015–25. 10.1681/ASN.2005080830 16565258

[42] Ma M Tian X Igarashi P Pazour GJ Somlo S. Loss of cilia suppresses cyst growth in genetic models of autosomal dominant polycystic kidney disease. Nat Genet (2013) 45:1004–12. 10.1038/ng.2715 23892607 PMC3758452

[43] Lee SH Somlo S. Cyst growth, polycystins, and primary cilia in autosomal dominant polycystic kidney disease. Kidney Res Clin Pract (2014) 33:73–8. 10.1016/j.krcp.2014.05.002 26877954 PMC4714135

[44] Delling M Indzhykulian AA Liu X Li Y Xie T Corey DP Primary cilia are not calcium-responsive mechanosensors. Nature (2016) 531:656–60. 10.1038/nature17426 27007841 PMC4851444

[45] Shibazaki S Yu Z Nishio S Tian X Thomson RB Mitobe M Cyst formation and activation of the extracellular regulated kinase pathway after kidney specific inactivation of Pkd1. Hum Mol Genet (2008) 17:1505–16. 10.1093/hmg/ddn039 18263604 PMC2902289

[46] Gallagher AR Germino GG Somlo S. Molecular advances in autosomal dominant polycystic kidney disease. Adv Chronic Kidney Dis (2010) 17:118–30. 10.1053/j.ackd.2010.01.002 20219615 PMC2837604

[47] Hopp K Ward CJ Hommerding CJ Nasr SH Tuan HF Gainullin VG Functional polycystin-1 dosage governs autosomal dominant polycystic kidney disease severity. J Clin Invest (2012) 122:4257–73. 10.1172/JCI64313 23064367 PMC3484456

[48] Fedeles SV Gallagher AR Somlo S. Polycystin-1: a master regulator of intersecting cystic pathways. Trends Mol Med (2014) 20:251–60. 10.1016/j.molmed.2014.01.004 24491980 PMC4008641

[49] Leonhard WN Happe H Peters DJ. Variable cyst development in autosomal dominant polycystic kidney disease: the biologic context. J Am Soc Nephrol (2016) 26:1322–33. 10.1681/ASN.2013080864 27493259 PMC5118495

[50] Lantinga-van Leeuwen IS Dauwerse JG Baelde HJ Leonhard WN van de Wal A Ward CJ Lowering of Pkd1 expression is sufficient to cause polycystic kidney disease. Hum Mol Genet (2004) 13:3069–77. 10.1093/hmg/ddh336 15496422

[51] Rossetti S Harris PC. Genotype-phenotype correlations in autosomal dominant and autosomal recessive polycystic kidney disease. J Am Soc Nephrol (2007) 18:1374–80. 10.1681/ASN.2006121387 17429049

[52] Pei Y Lan Z Wang K Garcia-Gonzalez M He N Dicks E A missense mutation in PKD1 attenuates the severity of renal disease. Kidney Int (2012) 81:412–7. 10.1038/ki.2011.370 22031115 PMC4105019

[53] Tan AY Blumenfeld J Michaeel A Donahue S Bobb W Parker T Autosomal dominant polycystic kidney disease caused by somatic and germline mosaicism. Clin Genet (2014) 87:373–7. 10.1111/cge.12383 24641620

[54] Fan LX Li X Magenheimer B Calvet JP Li X. Inhibition of histone deacetylases targets the transcription regulator Id2 to attenuate cystic epithelial cell proliferation. Kidney Int (2012) 81:76–85. 10.1038/ki.2011.296 21900881 PMC3409467

[55] Liu W Fan LX Zhou X Sweeney WE Jr Avner ED Li X. HDAC6 regulates epidermal growth factor receptor (EGFR) endocytic trafficking and degradation in renal epithelial cells. PLoS One (2012) 7:e49418. 10.1371/journal.pone.0049418 23152903 PMC3496684

[56] Zhou X Fan LX Sweeney WE Jr Denu JM Avner ED Li X. Sirtuin 1 inhibition delays cyst formation in autosomal-dominant polycystic kidney disease. J Clin Invest (2013) 123:3084–98. 10.1172/JCI64401 23778143 PMC4101988

[57] Zhou X Fan LX Li K Ramchandran R Calvet JP Li X. SIRT2 regulates ciliogenesis and contributes to abnormal centrosome amplification caused by loss of polycystin-1. Hum Mol Genet (2014) 23:1644–55. 10.1093/hmg/ddt556 24203696 PMC3929098

[58] Menezes LF Zhou F Patterson AD Piontek KB Krausz KW Gonzalez FJ Network analysis of a Pkd1-mouse model of autosomal dominant polycystic kidney disease identifies HNF4alpha as a disease modifier. PLoS Genet (2012) 8:e1003053 10.1371/journal.pgen.1003053 23209428 PMC3510057

[59] Bergmann C Von Bothmer J Ortiz Bruchle N Venghaus A Frank V Fehrenbach H Mutations in multiple PKD genes may explain early and severe polycystic kidney disease. J Am Soc Nephrol (2011) 22:2047–56. 10.1681/ASN.2010101080 22034641 PMC3279997

[60] Bergmann C. ARPKD and early manifestations of ADPKD: the original polycystic kidney disease and phenocopies. Pediatr Nephrol (2015) 30:15–30. 10.1007/s00467-013-2706-2 24584572 PMC4240914

[61] Brook-Carter PT Peral B Ward CJ Thompson P Hughes J Maheshwar MM Deletion of the TSC2 and PKD1 genes associated with severe infantile polycystic kidney disease – a contiguous gene syndrome. Nat Genet (1994) 8:328–32. 10.1038/ng1294-328 7894481

[62] Boddu R Yang C O’Connor AK Hendrickson RC Boone B Cui X Intragenic motifs regulate the transcriptional complexity of Pkhd1/PKHD1. J Mol Med (2014) 92:1045–56. 10.1007/s00109-014-1185-7 24984783 PMC4197071

[63] Lau EC Janson MM Roesler MR Avner ED Strawn EY Bick DP. Birth of a healthy infant following preimplantation PKHD1 haplotyping for autosomal recessive polycystic kidney disease using multiple displacement amplification. J Assist Reprod Genet (2010) 27:397–407. 10.1007/s10815-010-9432-5 20490649 PMC2922704

[64] Sweeney WE Jr Von Vigier RO Frost P Avner ED. Src inhibition ameliorates polycystic kidney disease. J Am Soc Nephrol (2008) 19:1331–41. 10.1681/ASN.2007060665 18385429 PMC2440293

[65] Gendreau SB Ventura R Keast P Laird AD Yakes FM Zhang W Inhibition of the T790M gatekeeper mutant of the epidermal growth factor receptor by EXEL-7647. Clin Cancer Res (2007) 13:3713–23. 10.1158/1078-0432.CCR-06-2590 17575237

[66] Trowe T Boukouvala S Calkins K Cutler RE Jr Fong R Funke R EXEL-7647 inhibits mutant forms of ErbB2 associated with lapatinib resistance and neoplastic transformation. Clin Cancer Res (2008) 14:2465–75. 10.1158/1078-0432.CCR-07-4367 18413839

[67] Shillingford JM Leamon CP Vlahov IR Weimbs T. Folate-conjugated rapamycin slows progression of polycystic kidney disease. J Am Soc Nephrol (2012) 23:1674–81. 10.1681/ASN.2012040367 22859856 PMC3458469

[68] Harskamp LR Gansevoort RT Boertien WE Van Oeveren W Engels GE Van Goor H Urinary EGF receptor ligand excretion in patients with autosomal dominant polycystic kidney disease and response to tolvaptan. Clin J Am Soc Nephrol (2015) 10:1749–56. 10.2215/CJN.09941014 26231191 PMC4594078

